# Early postoperative organ dysfunction is highly associated with the mortality risk of patients with type A aortic dissection

**DOI:** 10.1093/icvts/ivac266

**Published:** 2022-10-29

**Authors:** Ming-Hao Luo, Jing-Chao Luo, Yi-Jie Zhang, Xin Xu, Ying Su, Jia-Kun Li, Chun-Sheng Wang, Hao Lai, Yong-Xin Sun, Jun Li, Guo-Wei Tu, Zhe Luo

**Affiliations:** Shanghai Medical College, Fudan University, Shanghai, China; Department of Critical Care Medicine, Zhongshan Hospital, Fudan University, Shanghai, China; Department of Critical Care Medicine, Zhongshan Hospital, Fudan University, Shanghai, China; Department of Critical Care Medicine, Zhongshan Hospital, Fudan University, Shanghai, China; Department of Critical Care Medicine, Zhongshan Hospital, Fudan University, Shanghai, China; Department of Critical Care Medicine, Pan Long People’s Hospital, Kunming, China; Department of Critical Care Medicine, Zhongshan Hospital, Fudan University, Shanghai, China; Shanghai Medical College, Fudan University, Shanghai, China; Department of Critical Care Medicine, Zhongshan Hospital, Fudan University, Shanghai, China; Department of Cardiac Surgery, Zhongshan Hospital, Fudan University, Shanghai, China; Department of Cardiac Surgery, Zhongshan Hospital, Fudan University, Shanghai, China; Department of Cardiac Surgery, Zhongshan Hospital, Fudan University, Shanghai, China; Department of Cardiac Surgery, Zhongshan Hospital, Fudan University, Shanghai, China; Department of Critical Care Medicine, Zhongshan Hospital, Fudan University, Shanghai, China; Department of Critical Care Medicine, Zhongshan Hospital, Fudan University, Shanghai, China; Department of Critical Care Medicine, Zhongshan Hospital (Xiamen), Fudan University, Xiamen, China; Shanghai Key Lab of Pulmonary Inflammation and Injury, Shanghai, China

**Keywords:** Aortic dissection, Organ dysfunction, The Sequential Organ Failure Assessment, Mortality risk

## Abstract

**OBJECTIVES:**

This study assessed the impact of early postoperative organ dysfunction (EPOD) on in-hospital mortality of patients with type A aortic dissection (TAAD) after surgery.

**METHODS:**

Patients with TAAD who underwent surgical repair requiring deep hypothermic circulatory arrest from January 2020 to December 2021 were included. The Sequential Organ Failure Assessment (SOFA) score was calculated for 3 days postoperatively to stratify the severity of organ dysfunction. Patients with the SOFA of 0–4, 5–8 or >8 were defined as mild, moderate or severe EPOD. The primary outcome was in-hospital mortality, and a composite secondary outcome was defined as in-hospital death or any major complications. Kaplan–Meier curves were used to compare survival probability. The area under the receiver operating characteristic curve and calibration plots were used to evaluate the predictive power and overall performance of SOFA.

**RESULTS:**

Of the 368 patients, 5 patients (3%) with moderate EPOD and 33 patients (23%) with severe EPOD died. No patient died with mild EPOD. The areas under the receiver operating characteristic curve of SOFA for predicting mortality and the composite outcome were 0.85 (0.81–0.88) and 0.81 (0.77–0.85) on postoperative day 1. Each point of postoperative day 1 SOFA score corresponded to an odds ratio of 1.65 (1.42–1.92) for mortality. Of the 6 components of the SOFA system, only coagulation (2.34 [1.32–4.13]), cardiovascular (1.47 [1.04–2.08]), central nervous system (1.96 [1.36–2.82]) and renal (1.67 [1.04–2.70]) functions were associated with the higher risk of mortality.

**CONCLUSIONS:**

EPOD stratified by the SOFA score was associated with a higher risk of death and predicted the clinical outcomes of patients with TAAD with good accuracy.

**Figure:**
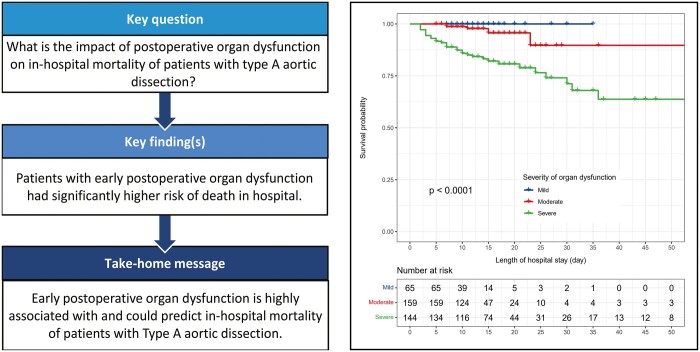

## INTRODUCTION

Appropriate risk stratification for patients with type A aortic dissection (TAAD) is important, especially for those who received surgical repair. A large number of studies have focused on preoperative evaluations to help with surgical decisions [1, 2], but despite advances in cardiothoracic surgery, mortality rates remain high after operations. A thorough postoperative assessment is of great necessity to tailor interventions to patient’s critical conditions to improve the prognosis.

Organ dysfunction typically manifests early in the postoperative period and can have lasting negative effects on patients. Early postoperative organ dysfunction (EPOD) not only accurately reflects the severity of the disease in the early postoperative period but also serves as an important intermediate factor in deciding the prognosis [3, 4]. Before definitive operations, a great number of patients have already experienced renal, hepatic, respiratory or cardiac dysfunction depending on the extent of the aortic tear [5]. In addition, multiple factors during surgery, such as operative trauma, prolonged surgery, cardiopulmonary bypass (CPB), deep hypothermic circulatory arrest (DHCA) and large volume of transfusion, combined with preoperative conditions, all contribute to the development or the worsening of severe organ dysfunction. In consideration of these issues, the need to clinically assess organ dysfunction to help with early risk stratification for TAAD patients undergoing surgery is urgent.

As EPOD often involves multiple organs, a more comprehensive approach is preferred. Scoring systems have been explored in the field of critical care medicine for many years. The Sequential Organ Failure Assessment (SOFA) score is a simple and common scoring system that allows for the calculation of the severity of organ dysfunction in 6 organ systems [6], and the score can measure individual or aggregate organ dysfunction [7]. It has been widely adopted to evaluate multiple organ dysfunction syndrome in septic patients [8, 9]. In addition, parameters used in the SOFA score are readily available clinically with good convenience. Therefore, we propose the hypothesis that EPOD measured by the SOFA score postoperatively may be associated with and could predict mortality and major adverse outcomes.

The aims of this study were to (i) assess the association and the predictive value of EPOD stratified by the SOFA score in the risk of death and major complications and (ii) evaluate the different impacts of different organ dysfunction on clinical prognosis in patients with TAAD after surgery.

## METHODS

### Ethical statement

The institutional ethics committee (Zhongshan Hospital, Fudan University) has approved this study (approval number: B2019-075R). Informed consent was waived due to the retrospective nature of the study.

### Study population

**Figure 1:**
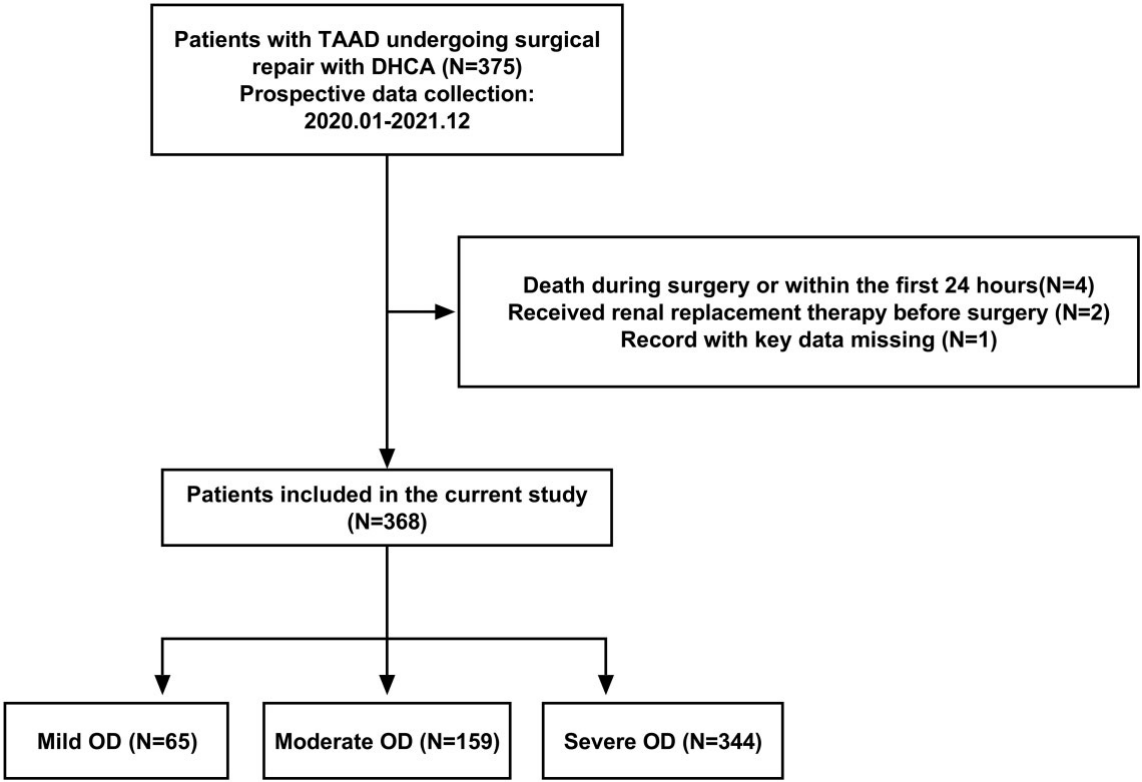
Flowchart of patients included in the study. DHCA: deep hypothermic circulatory arrest; OD: organ dysfunction; TAAD: type A aortic dissection.

This retrospective study was conducted in a 40-bed cardiac surgical intensive care unit in a university teaching hospital. Patients with TAAD who underwent surgical repair with DHCA at our centre between January 2020 and December 2021 were included. Participants meeting one of the following were excluded: (i) age < 18 years; (ii) death during surgery or within the first 24 h postoperatively; (iii) received renal replacement therapy before surgery; (v) confirmed neurological conditions by computer tomography (CT) or magnetic resonance imaging due to aortic dissection prior to surgery; and (vi) record with key data missing (Fig. 1).

### Surgical techniques

All procedures were performed through median sternotomy under total CPB with DHCA and selective cerebral perfusion. Venous drainage from the right atrium and peripheral arterial cannulation via the right axillary artery and femoral artery were initiated for CPB. Unilateral selective cerebral perfusion was started through the right axillary artery once the circulatory arrest was initiated with the brachiocephalic arteries cross-clamped and was conducted under the flow rate at 8–10 ml/kg. The perfusion state was adjusted under the guidance of near-infrared spectroscopy monitoring.

The particular surgical procedure was determined by the specific anatomic classification and the on-duty cardiac surgeon. Aortic arch repair was performed under a direct inspection and open anastomosis. The total arch replacement was conducted with either an *en bloc* style or a tertrafurcate conduit vascular graft. Once the distal anastomosis and the left common carotid artery reconstruction were completed, a systemic rewarming was started with bilateral cerebral and downstream aortic reperfusion. If aortic dissection extended beyond the distal arch, a stented elephant trunk was implanted into the distal aorta. In patients requiring hemiarch replacement, the lesser curvature of the aortic arch was resected. A bevelled incision was made for the distal end of a vascular graft to replace the arch.

### Data collection

During the study period, the perioperative information was obtained and stored in the institutional TAAD database. Laboratory examinations were performed at around 7 in the morning and physiological data, such as blood pressure, were collected at the same time. Preoperative demographic information and results of laboratory tests, surgical data and the incidence of major postoperative complications were also recorded. SOFA, quick SOFA(q-SOFA), Acute Physiology and Chronic Health Evaluation II and European System for Cardiac Operative Risk Evaluation II (EuroSCORE II) scores were then calculated by physicians on duty based on previous publications [7, 10–12]. Where individual components of certain scoring system were missing, multiple imputation was used. Where all components were unknown, the patient was excluded from the study.

### Outcomes

The primary outcome was all-cause in-hospital mortality. The second outcome was a composite defined as death or any of the following major adverse conditions: stroke, tracheotomy, reintubation, acute renal failure requiring renal replacement therapy, infection and gastrointestinal haemorrhage.

### Clinical definitions

EPOD was classified as mild, moderate or severe based on the SOFA score on postoperative day (POD) 1 (13–15). Mild EPOD was defined as having an SOFA score of 0–4. Moderate and severe EPOD was defined as having an SOFA score of 5–8 or above 8, respectively.

The diagnostic criteria for stroke were that patients appeared persistent unconscious (>6 h) or other neurological symptoms/signs after surgery, after excluding anaesthesia factors. Head CT plain scan and head CT perfusion imaging were implemented and neurologists were consulted to confirm the diagnosis of stroke.

Microbial cultivation was taken to confirm potential infection. When the result turned out to be negative, infection was still considered and diagnosed clinically based on clinical manifestations, radiological evidence, culture and biomarkers.

### Statistical analysis

Continuous variables were expressed as means and standard deviations for normally distributed values or medians and interquartile ranges (IQRs) (25th and 75th percentiles) for skewedly distributed values. Categorized variables were expressed as numbers and percentages. Data were compared with Student’s *t*-test or Wilcoxon rank-sum test or Fisher’s exact test. Radar chart was used to show the change in the severity of each organ dysfunction over the first 3 days. Kaplan–Meier curves and log-rank test were used to estimate hospital survival in patients with mild, moderate and severe EPOD. The logistic regression was used to calculate the odds ratio (OR) of each organ system for mortality or composite outcome. Receiver operating characteristic curves were generated and the areas under the receiver operating characteristic curves (AUROCs) were calculated to compare the predictive accuracies (deLong's test). Calibration plots, including the Brier score, were made to indicate overall model performance. In addition, decision analysis curve was depicted to analyse the net benefit of the SOFA score.

All statistical tests were two-sided and *P* < 0.05 was considered statistically significant. Statistical analyses were performed using R, version 4.2.1 (R Foundation for Statistical Computing, Vienna, Austria).

## RESULTS

### Baseline and perioperative characteristics

During the study period, 368 patients (mean age: 53; male: 74%) were finally included in the current study (Fig. 1). Their characteristics are summarized in Table 1. Of them, 65 had mild EPOD. A total of 159 experienced moderate EPOD and 144 underwent severe EPOD. Patients in all 3 groups had similar demographics and co-morbidities. In terms of preoperative laboratory values, those who were worse at organ function had significantly higher serum creatinine, aspartate aminotransferase, D-dimer and lower platelets. They also had a higher total bilirubin, alanine aminotransferase and cardiac troponin T.

**Table 1:** Patient demographics and perioperative data

	All patients (*n* = 368)	Mild OD (*n* = 65)	Moderate OD (*n* = 159)	Severe OD (*n* = 144)	*P*-Value
Age (years)	53 (13)	50 (14)	53 (12)	55 (13)	0.020
Male, *n* (%)	273 (74)	43 (66)	114 (72)	116 (81)	0.054
Body mass index (kg/m^2^)	26 (5)	24 (3)	26 (5)	27 (5)	0.002
Hypertension, *n* (%)	231 (63)	37 (57)	97 (61)	97 (67)	0.293
Diabetes mellitus, *n* (%)	16 (4)	1 (2)	6 (4)	9 (6)	0.323
Coronary heart disease, *n* (%)	13 (4)	5 (8)	4 (3)	4 (3)	0.159
Preoperative lab values					
Creatinine (μmol/l)	75 (63–97)	65 (54–76)	72 (61–87)	91 (72–123)	<0.001
WBC (10^9^/l)	11.6 (7.5)	11.7 (15.3)	10.8 (4.5)	12.3 (4.2)	0.221
Haemoglobin (g/l)	130 (22)	126 (26)	130 (20)	132 (21)	0.131
Platelets (10^9^/l)	173 (66)	208 (68)	175 (64)	155 (60)	<0.001
Bilirubin (μmol/l)	16 (9)	13 (8)	15 (7)	17 (10)	0.012
ALT (U/l)	26 (17–40)	23 (17–36)	23 (15–35)	30 (22–45)	0.001
AST (U/l)	25 (19–34)	22 (17–26)	23 (18–30)	29 (22–46)	<0.001
NT-pro BNP (pg/ml)	176 (86–543)	159 (82–300)	176 (66–504)	220 (119–627)	0.017
cTnT (ng/ml)	0.01 (0.01–0.03)	0.01 (0–0.02)	0.01 (0.01–0.03)	0.02 (0.01–0.06)	<0.001
D-dimer (mg/l)	6.6 (2.6–17.7)	3.2 (1.5–7.5)	5.5 (2.5–17.3)	11.9 (3.9–28.9)	<0.001
PT (s)	16.8 (15.8–17.8)	16.1 (15.3–17.4)	16.8 (15.8–17.6)	17.2 (16.1–18.5)	0.001
APTT (s)	27.3 (25.3–29.3)	27.3 (25.2–29.3)	27.3 (25.3–29.1)	27.5 (25.4–29.7)	0.664
Information on POD 1					
HR (beat/min)	90 (14)	89 (13)	90 (13)	91 (16)	0.622
SBP (mmHg)	111 (19)	112 (15)	116 (20)	104 (19)	<0.001
DBP (mmHg)	59 (11)	67 (10)	60 (11)	55 (10)	<0.001
MAP (mmHg)	76 (12)	80 (8)	79 (12)	71 (11)	<0.001
Lactate (mmol/l)	3.1 (2.0–4.7)	2.5 (1.9–3.5)	3.0 (2.0–4.2)	3.8 (2.3–7.7)	<0.001
Creatinine (μmol/l)	126 (86–170)	94 (81–108)	118 (92–145)	167 (128–228)	<0.001
WBC (10^9^/l)	11.6 (3.9)	11.4 (3.5)	11.5 (3.8)	11.7 (4.2)	0.885
Haemoglobin (g/l)	101 (15)	102 (14)	103 (15)	98 (16)	0.027
Platelets (10^9^/l)	94 (46)	135 (50)	96 (40)	74 (37)	<0.001
Bilirubin (μmol/l)	34 (18)	28 (17)	35 (18)	36 (19)	0.013
ALT (U/l)	20 (13–33)	16 (12–24)	18 (12–29)	22 (15–49)	<0.001
AST (U/l)	48 (37–68)	40 (31–48)	46 (36–60)	61 (43–121)	<0.001
NT-pro BNP (pg/ml)	607 (345–1213)	442 (237–777)	546 (301–1056)	840 (475–1570)	<0.001
cTnT (ng/ml)	0.48 (0.31–0.84)	0.31 (0.20–0.46)	0.43 (0.29–0.75)	0.73 (0.44–1.40)	<0.001
Risk scores					
EuroSCORE II	8 (6–9)	8 (6–9)	8 (6–9)	8 (6–9)	0.321
POD 1 APACHE II	14 (11–18)	11 (9–14)	13 (10–16)	18 (13–22)	<0.001
POD 1 q-SOFA	1 (0–1)	0 (0–1)	1 (0–1)	1 (1–2)	<0.001
POD 1 SOFA	7 (5–10)	3 (2–4)	6 (6–7)	11 (10–12)	<0.001
POD 2 SOFA	6 (4–9)	3 (2–4)	5 (4–7)	10 (8–13)	<0.001
POD 3 SOFA	4 (2–8)	2 (1–3)	3 (2–5)	8 (6–11)	<0.001
Clinical outcomes					
Stroke, *n* (%)	50 (14)	2 (3)	7 (4)	41 (29)	<0.001
Tracheostomy, *n* (%)	45 (12)	0 (0.0)	6 (4)	39 (27)	<0.001
Reintubation, *n* (%)	9 (2)	1 (2)	2 (1)	6 (4)	0.230
RRT, *n* (%)	54 (15)	1 (2)	6 (4)	47 (33)	<0.001
Gastrointestinal bleeding, *n* (%)	4 (1)	0 (0)	0 (0)	4 (3)	0.041
Infection, *n* (%)	28 (8)	0 (0)	3 (2)	25 (18)	<0.001
Death, *n* (%)	38 (10)	0 (0)	5 (3)	33 (23)	<0.001
Composite outcome, *n* (%)	103 (28)	3 (5)	17 (11)	83 (58)	<0.001
Length of MV (days)	2 (2–4)	1 (1–2)	2 (2–3)	4 (3–9)	<0.001
Length of ICU (days)	5 (3–8)	3 (2–4)	4 (3–6)	8 (5–15)	<0.001
Length of hospital stay (days)	13 (10–18)	11 (8–14)	12 (10–16)	15 (11–23)	<0.001

Values are represented as mean and standard deviation, median (interquartile range) or number (%).

WBC: white blood cell; ALT: alanine aminotransferase; APACHE II: Acute Physiology and Chronic Health Evaluation II; PT: prothrombin time; APTT: activated partial thromboplastin time; AST: aspartate aminotransferase; DBP: diastolic blood pressure; EuroSCORE II: European System for Cardiac Operative Risk Evaluation II; HR: heart rate; ICU: intensive care unit; MAP: mean arterial pressure; MV: mechanical ventilation; NT-pro BNP: N-terminal-pro B-type natriuretic peptide; cTnT: cardiac troppnin T; OD: organ dysfunction; POD: postoperative day; q-SOFA: quick SOFA; SBP: systolic blood pressure; SOFA: Sequential Organ Failure Assessment; RRT: renal replacement therapy.

Perioperative information concerning the type of surgical procedure, CPB and transfusion are shown in Supplementary Material, Table S1. Most patients underwent total aortic arch replacement with frozen elephant trunk technique. The mean CPB duration was 178 min and the mean duration of DHCA was 18 min. In addition, the mean lowest nasopharyngeal temperature and bladder temperature recorded during CPB were 22.3 and 26.1°C, respectively.

### Patients with severe early postoperative organ dysfunction had higher mortality

**Figure 2:**
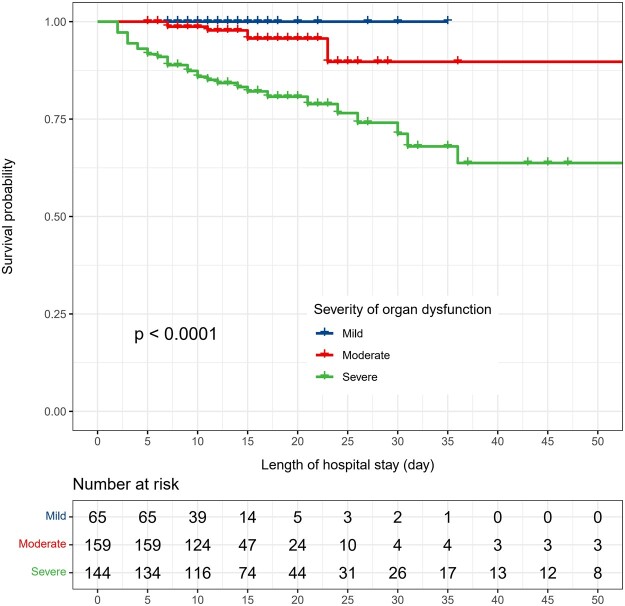
Kaplan–Meier analysis of type A aortic dissection patients with early postoperative organ dysfunction.

Throughout the entire hospital stay, a primary outcome event (death) had occurred in 5 of 368 patients (3%) in the moderate EPOD group and in 33 of 368 patients (23%) in the severe EPOD group. No patients died in the mild EPOD group (Table 1 and Fig. 2). Individual univariable logistic regressions were made to assess factors that might be associated with mortality. ORs of comorbidity, pre- and postoperative lab value and risk score are summarized in Supplementary Material, Table S2. Among them, the OR of POD 1 SOFA for morality was 1.65 (1.42–1.92) (*P* < 0.001).

### Failure of different organ systems correlated variously with prognosis

The ORs of each score of SOFA (POD 1) for mortality and the composite outcome were 1.65 [95% confidence interval (CI) 1.42–1.92] and 1.61 (95% CI 1.40–1.86). Patients who had a similar SOFA score overall but different scores for each category showed different risks of in-hospital mortality (Table 3). Improvements in different organ systems also varied throughout the early postoperative period (Supplementary Material, Fig. S1). Of the 6 components of the SOFA system, only coagulation (OR 2.34 [95% CI: 1.32–4.13]), cardiovascular (OR 1.47 [95% CI: 1.04–2.08]), central nervous system (CNS) (OR 1.96 [95% CI: 1.36–2.82]) and renal (OR 1.67 [95% CI: 1.04–2.70]) functions were associated with the higher risk of mortality. Different scores for each category in SOFA also indicated different risks for major adverse events. All but changes in the total bilirubin (OR, 1.03 (95% CI: 0.65–1.63), *P* = 0.891) were associated with the increased risk of major adverse events.

**Table 2:** The predictive accuracy of the Sequential Organ Failure Assessment, quick Sequential Organ Failure Assessment, Acute Physiology and Chronic Health Evaluation II and European System for Cardiac Operative Risk Evaluation II scores

Risk score	AUROC	Best cutoff	Sensitivity	Specificity
For mortality				
EuroSCORE II	0.61 (0.56–0.66)	8	50 (34–66)	69 (64–74)
POD 1 APACHE II	0.74 (0.69–0.78)	19	55 (38–71)	83 (78–87)
POD 1 q-SOFA	0.67 (0.62–0.72)	1	45 (29–62)	81 (77–85)
SOFA score				
POD 1	0.85 (0.81–0.88)	9	82 (66–92)	76 (71–81)
POD 2	0.85 (0.81–0.89)	10	71 (54–85)	86 (82–89)
POD 3	0.87 (0.83–0.90)	7	79 (63–90)	80 (76–85)
For the composite outcome				
EuroSCORE II	0.61 (0.56–0.66)	8	44 (34–54)	71 (65–76)
POD1 APACHE II	0.66 (0.61–0.71)	19	44 (30–59)	89 (84–92)
POD 1 q-SOFA	0.64 (0.58–0.69)	1	33 (20–48)	86 (82–90)
SOFA score				
POD 1	0.81 (0.77–0.85)	9	65 (50–78)	87 (82–91)
POD 2	0.93 (0.90–0.96)	7	100 (59–100)	76 (70–80)
POD 3	0.85 (0.81–0.89)	5	764 (57–90)	81 (76–86)

Data are presented as true value (95% CI).

APACHE II: Acute Physiology and Chronic Health Evaluation II; AUROC: areas under the receiver operating characteristic curves; CI: confidence interval; EuroSCORE II: European System for Cardiac Operative Risk Evaluation II; POD: postoperative day; SOFA: Sequential Organ Failure Assessment; q-SOFA: quick SOFA.

**Table 3:** Odds ratios for in-hospital mortality and for the composite outcome with per unit increase in each scoring system

Risk score	For mortality		For the composite outcome	
	OR (95% CI)	*P*-Value	OR (95% CI)	*P-*Value
EuroSCORE II	1.19 (1.05–1.36)	0.009	1.17 (1.07–1.27)	<0.001
POD 1 APACHE II	1.15 (1.10–1.21)	<0.001	1.14 (1.07–1.21)	<0.001
POD 1 q-SOFA	2.26 (1.47–3.49)	<0.001	1.95 (1.29–2.94)	0.001
POD 1 SOFA	1.65 (1.42–1.92)	<0.001	1.61 (1.40–1.86)	<0.001
Respiration	1.37 (0.83–2.26)	0.223	1.62 (1.01–2.59)	0.045
Coagulation	2.34 (1.32–4.13)	0.004	1.75 (1.07–2.85)	0.025
Liver	0.92 (0.56–1.51)	0.751	1.03 (0.65–1.63)	0.891
Cardiovascular	1.47 (1.04–2.08)	0.030	1.33 (1.01–1.74)	0.041
Central nervous system	1.96 (1.36–2.82)	<0.001	2.72 (1.69–4.37)	<0.001
Renal	1.67 (1.04–2.70)	0.035	1.88 (1.17–3.04)	0.010

Data are presented as true value (95% CI).

APACHE II: Acute Physiology and Chronic Health Evaluation II; CI: confidence interval; EuroSCORE II: European System for Cardiac Operative Risk Evaluation II; OR: odds ratio; POD: postoperative day; SOFA: Sequential Organ Failure Assessment; q-SOFA: quick SOFA.

### Early postoperative organ dysfunction could well predict mortality and the composite outcome

**Figure 3:**
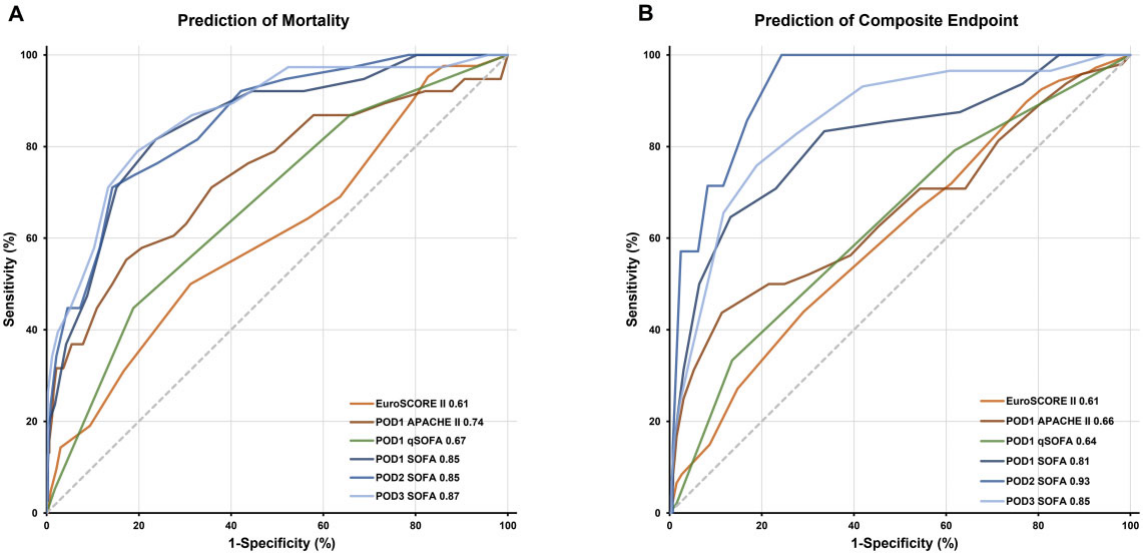
Receiver operating characteristic curves of 4 scoring systems for in-hospital all-cause mortality and the composite outcome. (**A**) Mortality prediction. POD 1 SOFA (AUROC, 0.85 [95% CI: 0.81–0.88]), POD 2 SOFA (AUROC, 0.85 [95% CI: 0.81–0.89]), POD 3 SOFA (AUROC, 0.87 [95% CI: 0.83–0.90]), POD 1 q-SOFA (AUROC, 0.67 [95% CI: 0.62–0.72]), POD 1 APACHE II (AUROC 0.74, [95% CI: 0.69–0.78]), EuroSCORE II (AUROC, 0.61 [95% CI: 0.56–0.66]). (**B**) Prediction of the composite outcome. POD 1 SOFA (AUROC, 0.81 [95% CI: 0.77–0.85]), POD 2 SOFA (AUROC, 0.93 [95% CI: 0.90–0.96]), POD 3 SOFA (AUROC, 0.85 [95% CI: 0.81–0.89]), POD 1 q-SOFA (AUROC, 0.64 [95% CI: 0.58–0.69]), POD 1 APACHE II (AUROC, 0.66 [95% CI: 0.61–0.71]), POD 1 EuroSCORE II (AUROC, 0.61 [95% CI: 0.56–0.66]). APACHE II: Acute Physiology and Chronic Health Evaluation II; AUROC: areas under the receiver operating characteristic curves; CI: confidence interval; EuroSCORE II: European System for Cardiac Operative Risk Evaluation II; POD: postoperative day; SOFA: Sequential Organ Failure Assessment; q-SOFA: quick SOFA.

On POD 1, mild EPOD patients had a mean SOFA score of 3 [IQR 2–4], while patients with moderate and severe EPOD had a mean SOFA score of 6 [IQR 6–7] and 11 [IQR 10–12] respectively (Table 1). The AUROCs of POD 1 SOFA for mortality and the composite outcome were 0.85 (95% CI: 0.81–0.88) and 0.81 (95% CI: 0.77–0.85). The SOFA score then declined in all groups but remained high in patients with severe EPOD (POD 2: 3 [IQR 2–4] vs 5 [IQR 4–7] vs 10 [IQR 8–13], *P* < 0.001; POD 3: 2 [IQR 1–3] vs 3 [IQR 2–5] vs 8 [IQR 6–11], *P* < 0.001). It also maintained its AUROC above 0.85 throughout the early postoperative period for both mortality and composite outcome prediction [POD 2, AUROC, 0.85 (95% CI: 0.81–0.89), 0.93 (95% CI: 0.90–0.96); POD 3, AUROC, 0.87 (95% CI: 0.83–0.90), 0.85 (95% CI: 0.81–0.89)] (Fig. 3 and Table 1).

A cutoff value of 9 on POD 1 [sensitivity, 82 (95% CI: 66–92), specificity, 76 (95% CI: 71–81)], 10 on POD 2 [sensitivity, 71 (95% CI: 54–85), specificity, 86 (95% CI: 82–89)] and 7 on POD 3 [sensitivity, 79 (95% CI: 63–90), specificity, 80 (95% CI: 76–85)] for SOFA score was defined to predict in-hospital mortality (Table 2).

The calibration plots of different scoring systems can be found in Supplementary Material, Figs S2 and S3. The overall performances of SOFA were still good, but less outstanding than its discriminative power (Brier scores of 0.071, 0.071 and 0.066 on POD 1, 2 and 3, respectively), which implies that a higher SOFA score on POD 1 might overestimate mortality risk, but such an overestimation was gradually made up in the following days (Supplementary Material, Figs S2 and S3).

### Sequential Organ Failure Assessment had better predictive accuracy than that of Acute Physiology and Chronic Health Evaluation II and European System for Cardiac Operative Risk Evaluation II

AUROCs of EuroSCORE II for mortality and the composite outcome were much lower, at 0.61 (95% CI: 0.56–0.66) and 0.61 (95% CI: 0.56–0.66). The same applied to POD 1 Acute Physiology and Chronic Health Evaluation II at 0.74 (95% CI: 0.69–0.78) and 0.66 (95% CI: 0.61–0.71) (Fig. 3 and Table 2). The comparison among the AUCs showed a significant difference between the SOFA score and the EuroSCORE II (*P* < 0.001) (Supplementary Material, Table S3). A simplified version of SOFA, q-SOFA, was also less satisfactory. POD 1 q-SOFA showed less discriminatory power [AUROC, 0.67 (95% CI: 0.62–0.72)], with basically no improvements in the following 2 days (Fig. 3 and Table 2).

Decision curve analysis was made and is shown in Supplementary Material, Fig. S4. The net benefit was plotted against the threshold probability. The ‘all’ line showed the net benefit by evaluating all patients, and the ‘none’ line was the net benefit for evaluating no patients. It appeared that POD 1, 2 and 3 SOFA scores were always superior to the EuroSCORE II model across a wide range of threshold probabilities, with the highest difference at a threshold probability between 0.1 and 0.2. At that threshold, the net benefit was 0.06 for the SOFA score and 0.01 for EuroSCORE II.

## DISCUSSION

In this single-centre, retrospective study involving 368 patients with TAAD who underwent surgical repair requiring DHCA, EPOD measured by the SOFA score was associated with a higher risk of death and was a good predictor for in-hospital mortality and major adverse outcomes. The SOFA score demonstrated better discriminative power for both in-hospital mortality and major adverse outcomes compared to other scoring systems. Noticeably, not only the total of SOFA was important but also the organ from which scores were derived might affect patient outcomes. Changes in CNS, renal, cardiovascular and coagulation systems showed higher risk towards mortality, highlighting the focus of postoperative management.

Organ dysfunction is relatively common and occurs early in TAAD patients, owing to factors both pre- and intraoperatively, such as malperfusion. But a lack of tools for systematical evaluation throughout the perioperative period results in uncertainties about its impact on clinical outcomes. Previously, some studies with smaller sample sizes have highlighted the SOFA score as a risk factor for postoperative mortality [13]. Our study has indicated that simply counting the SOFA score as a risk factor undermines its great potential as a direct metric for prognosis prediction and overlooks its clinical significance in assessing organ dysfunction in TAAD patients after surgery. Although the SOFA score may overestimate mortality initially, repeated monitoring throughout the early postoperative period still could offer good results, emphasizing the importance of dynamic evaluation of organ functions after surgery. In addition, the net benefit of the SOFA score was higher than that of the EuroSCORE II at a threshold probability between 0.1 and 0.2, a range consistent with the mortality rate of patients with TAAD after surgery. Moreover, the SOFA score has been already widely used in intensive care units among patients with sepsis to assess organ dysfunction and achieved good results [6, 7], making it extremely easy and convenient for intensivists to adopt.

One of the main reasons why postoperative organ dysfunction develops can be the malperfusion syndrome (MS) throughout the perioperative period. MS occurs in around 25–30% of TAAD patients despite improvements in medical therapy and is an independent risk factor for poor prognosis [14]. In the current study, though patients receiving renal replacement therapy and experiencing neurological conditions due to aortic dissection were excluded, significant differences were found in terms of serum creatinine, platelets and total bilirubin between survivors and non-survivors before operations, indicating that MS might had occurred before surgery. Previously, Czerny *et al.* proposed the German Registry for Acute Type A Aortic Dissection score as a novel scoring system to predict the 30-day mortality rate for patients undergoing surgery for TAAD [15]. However, it was challenged for a lack of parameters to define organ malperfusion, considering the importance of it in patients with TAAD [16]. Instead, the SOFA score could provide physicians with parameters to monitor organ functions and further quantify the extent of MS. A high SOFA score in the early postoperative period has good discriminatory power for both mortality and major complications.

Another reason why postoperative organ dysfunction takes place may be overwhelming inflammation. TAAD patients undergoing surgery are exposed to a great number of factors predisposed them to inflammation, such as prolonged surgery, median sternotomy with extensive tissue damage, CPB, DHCA and large amount of transfusion. More importantly, TAAD patients meeting all 4 criteria of severe systemic inflammatory response syndrome after surgery had an increase in the likelihood of major postoperative major adverse events, including death [17]. As tools that effectively diagnose perioperative inflammation and accurately predict outcomes in patients at increased risk for systemic inflammation and subsequent organ failure are still under development [18], the SOFA score, a score which has already been used to evaluate organ dysfunction in diseases caused by dysregulated inflammation, can be a good option. And further studies need to be carried out, as the relationship between the extent of inflammation (using inflammatory markers to reflect) and the SOFA score has not been clearly explicated in the current study.

### Limitations

Limitations of this study include its single centre and retrospective nature. In addition, TAAD patients who underwent surgery without DHCA were not included in the study. Comparisons with a number of novel scoring systems, such as the German Registry for Acute Type A Aortic Dissection score, were not made due to data missing. Although the SOFA score postoperatively has been shown to be a good predictor for in-hospital mortality and major adverse outcomes, differences in the SOFA score may have already existed prior to surgery and further validations with larger sample sizes are still preferable.

## CONCLUSIONS

The severity of EPOD stratified by the SOFA score was associated with the higher risk of death in patients with TAAD and could be used to predict in-hospital mortality and major adverse events with good accuracy. Of the 6 components of the SOFA system, only coagulation, cardiovascular, CNS and renal functions were associated with the higher risk of mortality.

## SUPPLEMENTARY MATERIAL

Supplementary material is available at *ICVTS* online.

## Funding

This research was funded by the Smart Medical Care of Zhongshan Hospital (2020ZHZS01), the Science and Technology Commission of Shanghai Municipality (20DZ2261200), the National Natural Science Foundation of China (82070085), the Clinical Research Project of Zhongshan Hospital (2020ZSLC38 and 2020ZSLC27), the Project for Elite Backbone of Zhongshan Hospital (2021ZSGG06) and the Research Project of Shanghai Municipal Health Commission (20214Y0136).

**Conflict of interest:** none declared.

## Supplementary Material

ivac266_Supplementary_Data

## Data Availability

The data underlying this article cannot be shared publicly due to institutional data regulations.

## ABBREVIATIONS

AUROCs: Areas under the receiver operating characteristic curves
CI: Confidence interval
CNS: Central nervous system
CPB: Cardiopulmonary bypass
CT: Computer tomography
DHCA: Deep hypothermic circulatory arrest
EPOD: Early postoperative organ dysfunction
EuroSCORE II: European System for Cardiac Operative Risk Evaluation II
IQRs: Interquartile ranges
MS: Malperfusion syndrome
OR: Odds ratio
POD: Postoperative day
q-SOFA: Quick SOFA
SOFA: The Sequential Organ Failure Assessment
TAAD: Type A aortic dissection

## References

[1] Luo J-C , Zhong J, Duan W-X, Tu G-W, Wang C-S, Sun Y-X et al Early risk stratification of acute type A aortic dissection: development and validation of a predictive score. Cardiovasc Diagn Ther 2020;10:1827–38. 33381427 10.21037/cdt-20-730 PMC7758751

[2] Hou J-Y , Wang C-S, Lai H, Sun Y-X, Li X, Zheng J-L et al Veno-arterial extracorporeal membrane oxygenation for patients undergoing acute type A aortic dissection surgery: a six-year experience. Front Cardiovasc Med 2021;8:652527. 34079828 10.3389/fcvm.2021.652527 PMC8165157

[3] Olsson C , Franco-Cereceda A. Impact of organ failure and major complications on outcome in acute type A aortic dissection. Scand Cardiovasc J 2013;47:352–8. 24131200 10.3109/14017431.2013.845307

[4] Livingston D , Deitch E. Multiple organ failure: a common problem in surgical intensive care unit patients. Ann Med 1995;27:13–20. 7741992 10.3109/07853899509031931

[5] Yang B , Patel HJ, Williams DM, Dasika NL, Deeb GM. Management of type A dissection with malperfusion. Ann Cardiothorac Surg 2016;5:265–74. 27563540 10.21037/acs.2016.07.04 PMC4973125

[6] Jones AE , Trzeciak S, Kline JA. The Sequential Organ Failure Assessment score for predicting outcome in patients with severe sepsis and evidence of hypoperfusion at the time of emergency department presentation. Crit Care Med 2009;37:1649–54. 19325482 10.1097/CCM.0b013e31819def97 PMC2703722

[7] Vincent JL , Moreno R, Takala J, Willatts S, De Mendonça A, Bruining H et al The SOFA (Sepsis-related Organ Failure Assessment) score to describe organ dysfunction/failure. On behalf of the Working Group on Sepsis-Related Problems of the European Society of Intensive Care Medicine. Intensive Care Med 1996;22:707–10. 8844239 10.1007/BF01709751

[8] Vincent J , de Mendonça A, Cantraine F, Moreno R, Takala J, Suter P et al Use of the SOFA score to assess the incidence of organ dysfunction/failure in intensive care units: results of a multicenter, prospective study. Working group on "sepsis-related problems" of the European Society of Intensive Care Medicine. Crit Care Med 1998;26:1793–800. 9824069 10.1097/00003246-199811000-00016

[9] Soo A , Zuege D, Fick G, Niven D, Berthiaume L, Stelfox H et al Describing organ dysfunction in the intensive care unit: a cohort study of 20,000 patients. Crit Care 2019;23:186. 31122276 10.1186/s13054-019-2459-9 PMC6533687

[10] Seymour CW , Liu VX, Iwashyna TJ, Brunkhorst FM, Rea TD, Scherag A et al Assessment of clinical criteria for sepsis: for the Third International Consensus Definitions for Sepsis and Septic Shock (Sepsis-3). JAMA 2016;315:762–74. 26903335 10.1001/jama.2016.0288 PMC5433435

[11] Knaus WA , Draper EA, Wagner DP, Zimmerman JE. APACHE II: a severity of disease classification system. Crit Care Med 1985;13:818–29. 3928249

[12] Nashef SAM , Roques F, Sharples LD, Nilsson J, Smith C, Goldstone AR et al EuroSCORE II. Eur J Cardiothorac Surg 2012;41:744–5. 10.1093/ejcts/ezs043 22378855

[13] Huo Y , Zhang H, Li B, Zhang K, Li B, Guo S-H et al Risk factors for postoperative mortality in patients with acute Stanford type A aortic dissection. Int J Gen Med 2021;14:7007–15. 34707392 10.2147/IJGM.S330325 PMC8544269

[14] Crawford TC , Beaulieu RJ, Ehlert BA, Ratchford EV, Black JH. Malperfusion syndromes in aortic dissections. Vasc Med 2016;21:264–73. 26858183 10.1177/1358863X15625371 PMC4876056

[15] Czerny M , Siepe M, Beyersdorf F, Feisst M, Gabel M, Pilz M et al Prediction of mortality rate in acute type A dissection: the German Registry for Acute Type A Aortic Dissection score. Eur J Cardiothorac Surg 2020;58:700–6. 32492120 10.1093/ejcts/ezaa156

[16] Nežić DG , Živković IS, Miličić MD, Milačić PA, Košević DN, Boričić MI et al On-line risk prediction models for acute type A aortic dissection surgery: validation of the German Registry of Acute Aortic Dissection Type A score and the European System for Cardiac Operative Risk Evaluation II. Eur J Cardiothorac Surg 2022;61:1068–75. 34915555 10.1093/ejcts/ezab517

[17] Li J , Yang L, Wang G, Wang Y, Wang C, Shi S. Severe systemic inflammatory response syndrome in patients following total aortic arch replacement with deep hypothermic circulatory arrest. J Cardiothorac Surg 2019;14:217. 31842939 10.1186/s13019-019-1027-3 PMC6916067

[18] Margraf A , Ludwig N, Zarbock A, Rossaint J. Systemic inflammatory response syndrome after surgery: mechanisms and protection. Anesth Analg 2020;131:1693–707. 33186158 10.1213/ANE.0000000000005175

