# Supplementary material for: Early postoperative organ dysfunction is highly associated with the mortality risk of patients with type A aortic dissection
Source: Interact Cardiovasc Thorac Surg. 2022 Oct 29;35(6):ivac266. doi: 10.1093/icvts/ivac266 (PMC9642332; doi:10.1093/icvts/ivac266)
Supplement: ivac266_Supplementary_Data [file ivac266_supplementary_data.docx]

**Content**

Figure S1

Figure S2

Figure S3

Figure S4

Table S1

Table S2


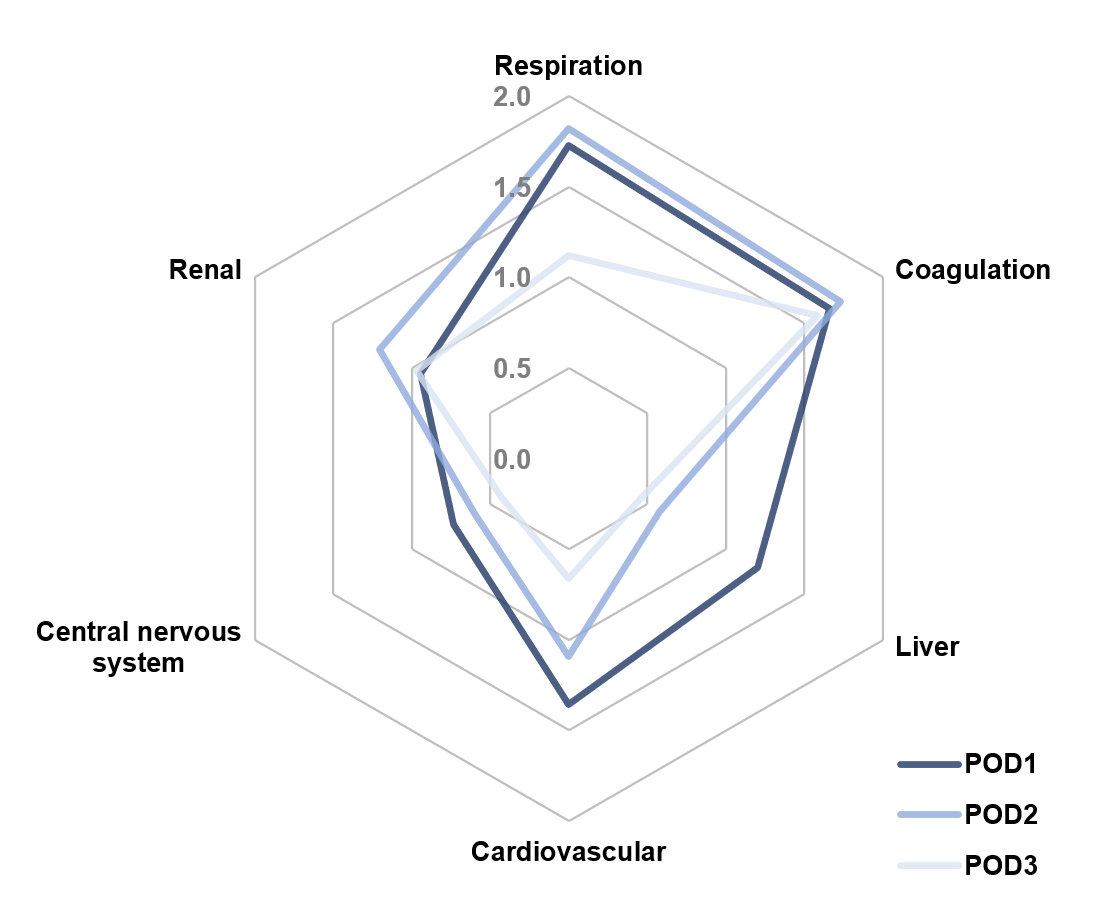


**Figure S1. Changes in mean values of six components of the SOFA score in the first three postoperative days**

SOFA= Sequential Organ Failure Assessment; POD=postoperative day


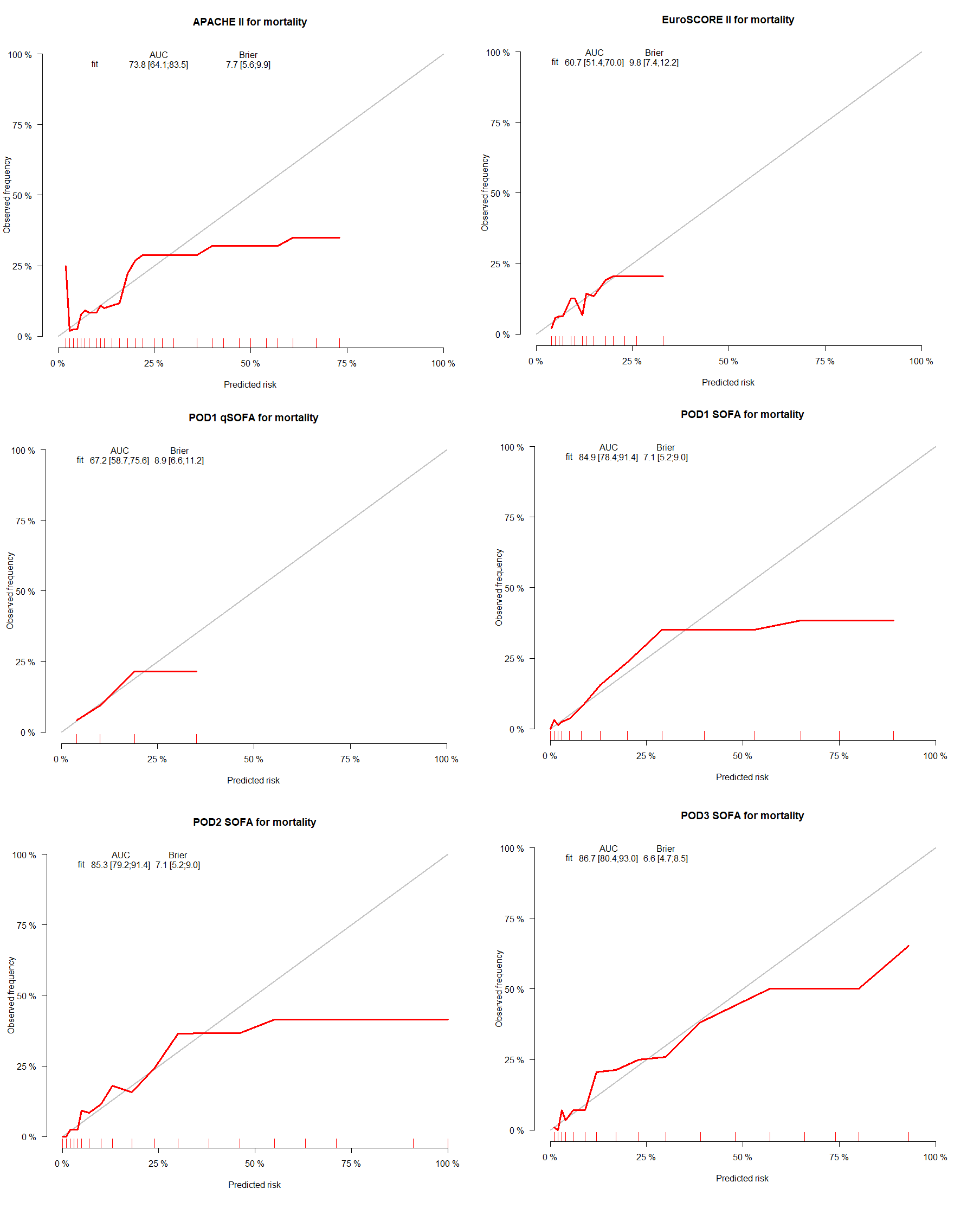


**Figure S2. Calibration curves for mortality**

APACHE II=Acute Physiology and Chronic Health Evaluation II; EuroSCORE II=European System for Cardiac Operative Risk Evaluation II; SOFA= Sequential Organ Failure Assessment; qSOFA= quick SOFA; POD=postoperative day


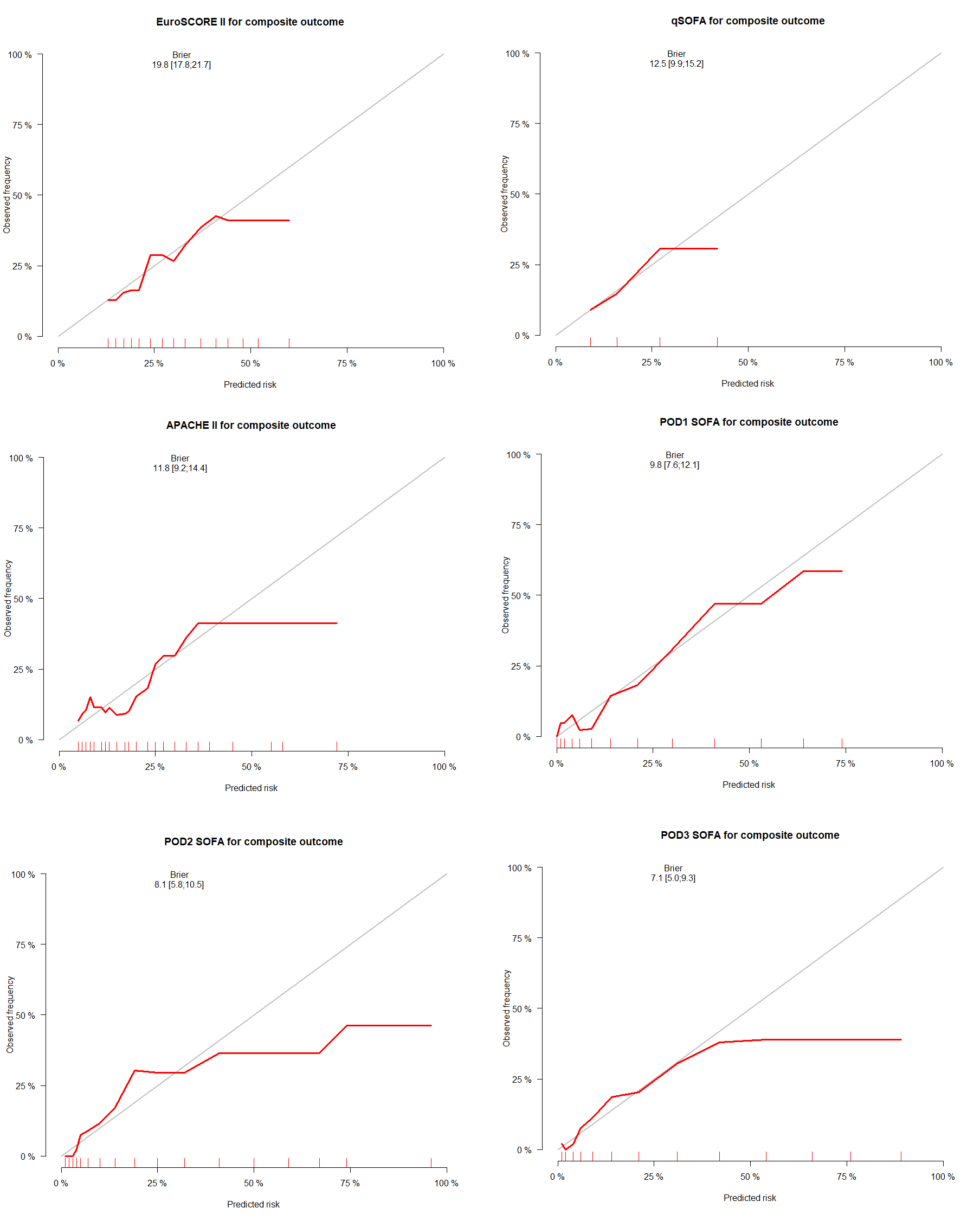


**Figure S3. Calibration curves for the composite outcome**

APACHE II=Acute Physiology and Chronic Health Evaluation II; EuroSCORE II=European System for Cardiac Operative Risk Evaluation II; SOFA= Sequential Organ Failure Assessment; qSOFA= quick SOFA; POD=postoperative day


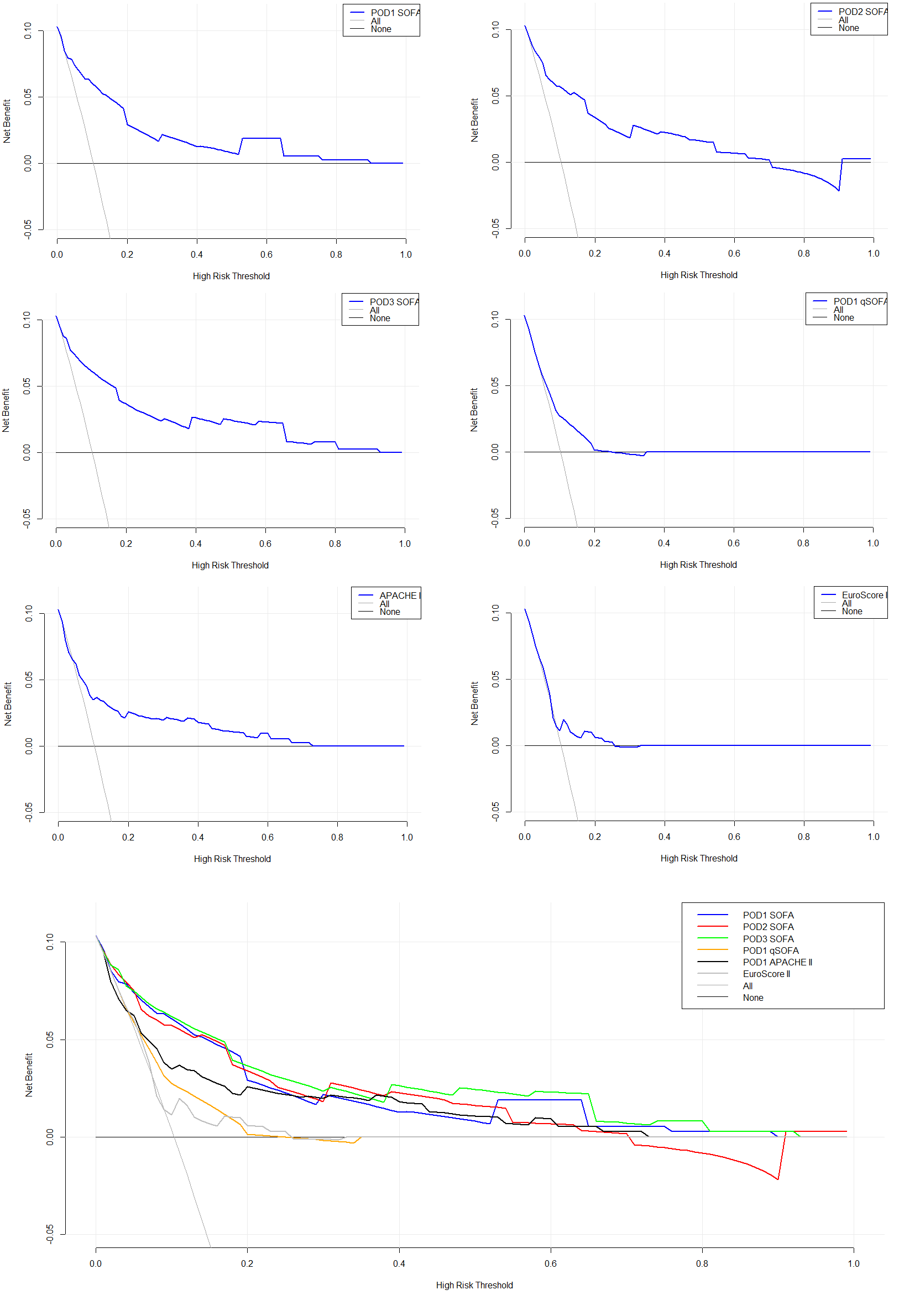


**Figure S4. Decision curve analysis for mortality risk**

APACHE II=Acute Physiology and Chronic Health Evaluation II; EuroSCORE II=European System for Cardiac Operative Risk Evaluation II; SOFA= Sequential Organ Failure Assessment; qSOFA= quick SOFA; POD=postoperative day

**Table S1 Operative information**

|  | All patients  (n=368) |
| --- | --- |
| **Surgical procedures** |  |
| Hemiarch replacement and concomitant procedures, n (%) | 26(7.1) |
| TAAR alone, n (%) | 5(1.4) |
| TAAR with FET, n (%) | 200(54.3) |
| TAAR with FET and Bentall, n (%) | 27(7.3) |
| TAAR with FET and David, n (%) | 27(7.3) |
| TAAR with FET and CABG, n (%) | 9(2.4) |
| **Operative Variables** |  |
| CPB time, mins | 178(152, 209) |
| Aortic cross clamp time, mins | 96(79, 120) |
| Parallel circulation time, mins | 63(49, 79) |
| DHCA time, min | 18(16, 23) |
| Lowest NPT, ℃ | 22.3(22.0, 23.1) |
| Lowest BT, ℃ | 26.1(25.5, 27.0) |
| **Transfusion** |  |
| Red blood cell, units | 4(0, 6) |
| Fresh frozen plasma, mL | 600(0, 800) |

Values are mead±standard deviation, median (interquartile range) or number (%)

TAAR=total aortic arch replacement; FET=frozen elephant trunk; CABG=coronary artery bypass grafting; CPB: cardiopulmonary bypass; DHCA: deep hypothermic circulatory arrest; NPT: nasopharyngeal temperature; BT: bladder temperature

**Table S2 Risk factors associated with postoperative mortality**

|  | Deceased (n=38) | Survived (n=330) | OR (95% CI) | p value |
| --- | --- | --- | --- | --- |
| Age, year | 56(15) | 53(13) | 1.02(0.99-1.05) | 0.202 |
| Male, n (%) | 31(82) | 242(73) | 1.61(0.68-3.79) | 0.275 |
| Body mass index (kg/m^2^) | 27(5 | 26(4 | 1.04(0.97-1.11) | 0.323 |
| Hypertension, n (%) | 26(68) | 205(62) | 1.32(0.64-2.71) | 0.448 |
| Diabetes mellitus, n (%) | 4(11) | 12(4) | 3.12(0.95-10.2) | 0.085 |
| Coronary heart disease, n (%) | 1(3) | 12(4) | 0.72(0.09-5.67) | 0.741 |
| Preoperative lab values |  |  |  |  |
| Creatinine, umol/L | 100(74-121) | 74(62-95) | 1.0084(1.0026-1.0142) | 0.004 |
| WBC, 10^9^/L | 13(4.8) | 11.4(7.8) | 1.02(0.99-1.05) | 0.215 |
| Hemoglobin, g/L | 128(22) | 131(22) | 0.9946(0.9798-1.0097) | 0.482 |
| Platelets, 109/L | 136(62) | 177(65) | 0.99(0.98-0.99) | <0.001 |
| Bilirubin, umol/L | 20(13) | 15(8) | 1.04(1.01-1.08) | 0.005 |
| ALT, U/L | 30(22-55) | 26(17-38) | 1.0009(0.9997-1.0021) | 0.132 |
| AST, U/L | 32(236- 69) | 24(18- 32) | 1.0019(1.0001-1.0036) | 0.035 |
| NT-pro BNP, pg/mL | 250(126- 860) | 174(83- 527) | 1.0002(0.9999-1.0004) | 0.189 |
| cTnT, ng/mL | 0.02(0.01- 0.10) | 0.01(0.01- 0.03) | 2.75(1.10-6.84) | 0.030 |
| D-dimer, mg/L | 17.8(4.7- 36.6) | 5.9(2.4- 16.7) | 1.04(1.02-1.07) | <0.001 |
| PT, s | 17.1(15.9- 18.1) | 16.80(15.8- 17.8) | 1.05(0.98-1.13) | 0.145 |
| APTT, s | 27.8(26.6- 31.4) | 27.3(25.3- 29.2) | 1.10(1.03-1.17) | 0.003 |
| Information on POD 1 |  |  |  |  |
| HR, beat/min | 93(17) | 90(14) | 1.02(0.99-1.04) | 0.154 |
| SBP, mmHg | 104(28) | 112(18) | 0.98(0.96-1.00) | 0.021 |
| DBP, mmHg | 56(12) | 59(10) | 0.97(0.94-1.00) | 0.067 |
| MAP, mmHg | 72(16) | 77(11) | 0.96(0.94-0.99) | 0.016 |
| Lactate, mmol/L | 5.1(3.1-9.3) | 3.0(2.0-4.50) | 1.16(1.09-1.24) | <0.001 |
| Creatinine, umol/L | 185(135-255) | 122(94-165) | 1.0098(1.0057-1.0138) | <0.001 |
| WBC, 10^9^/L | 11.3( 5.8) | 11.6( 3.6) | 0.98(0.90-1.07) | 0.636 |
| Hemoglobin, g/L | 96(17) | 102(15) | 0.97(0.95-1.00) | 0.020 |
| Platelets, 10^9^/L | 59(31) | 98(46) | 0.97(0.95-0.98) | <0.001 |
| Bilirubin, umol/L | 36(23) | 34(18) | 1.0044(0.9867-1.0225) | 0.626 |
| ALT, U/L | 25(19-93) | 18(13-31) | 1.0044(0.9867-1.0225) | 0.626 |
| AST, U/L | 69(46-207) | 47(36-66) | 1.0012(1.0003-1.0020) | 0.006 |
| NT-pro BNP, pg/mL | 1064(544-1895) | 590(336-1140) | 1.0002(1.0000-1.0003) | 0.004 |
| cTnT, ng/mL | 0.74(0.46-1.48) | 0.46(0.30-0.81) | 1.21(1.07-1.37) | 0.003 |
| Risk scores |  |  |  |  |
| EuroSCORE II | 9(6-10) | 8(6-9) | 1.18(1.04-1.34) | 0.009 |
| POD 1 APACHE II | 21(15-31) | 13(10-18) | 1.15(1.10-1.21) | <0.001 |
| POD 1 qSOFA | 1(1-2) | 1(0-1) | 2.26(1.47-3.49) | <0.001 |
| POD 1 SOFA | 11(10-13) | 7(5-9) | 1.65(1.42-1.92) | <0.001 |
| POD 2 SOFA | 12(9-15) | 6(4-8) | 1.41(1.27-1.56) | <0.001 |
| POD 3 SOFA | 11(8-15) | 4(2-6) | 1.45(1.31-1.61) | <0.001 |

Values are mean and standard deviation, median (interquartile range) or number (%)

OR=odds ratio; ALT= alanine aminotransferase; AST=aspartate aminotransferase; NT-pro BNP=N-terminal-pro B-type natriuretic peptide; APTT=activated partial thromboplastin time; HR=heart rate; SBP=systolic blood pressure; DBP=diastolic blood pressure; MAP=mean arterial pressure; APACHE II=Acute Physiology and Chronic Health Evaluation II; EuroSCORE II=European System for Cardiac Operative Risk Evaluation II; SOFA= Sequential Organ Failure Assessment; qSOFA= quick SOFA; POD=postoperative day; RRT=renal replacement therapy; MV=mechanical ventilation; ICU= intensive care unit

**Table S3. p-value for AUROC comparisons**

| Endpoints | Risk score | POD1  APACHE II | EuroSCORE II | POD1  SOFA | POD2  SOFA | POD3  SOFA |
| --- | --- | --- | --- | --- | --- | --- |
| Mortality | POD1 qSOFA | 0.245 | 0.442 | <0.001 | <0.001 | <0.001 |
|  | POD3 SOFA | 0.012 | <0.001 | 0.549 | 0.437 |  |
|  | POD2 SOFA | 0.020 | <0.001 | 0.857 |  |  |
|  | POD1 SOFA | 0.375 | <0.001 |  |  |  |
|  | EuroSCORE II | 0.104 |  |  |  |  |
| Composite outcome | POD1 qSOFA | 0.700 | 0.128 | <0.001 | <0.001 | <0.001 |
|  | POD3 SOFA | <0.001 | <0.001 | 0.021 | 0.074 |  |
|  | POD2 SOFA | <0.001 | <0.001 | 0.201 |  |  |
|  | POD1 SOFA | 0.007 | <0.001 |  |  |  |
|  | POD1 EuroSCORE II | 0.021 |  |  |  |  |

APACHE II=Acute Physiology and Chronic Health Evaluation II; EuroSCORE II=European System for Cardiac Operative Risk Evaluation II; SOFA= Sequential Organ Failure Assessment; qSOFA= quick SOFA; POD=postoperative day
